# Supplementary material for: Membrane Properties of Human Induced Pluripotent Stem Cell-Derived Cultured Red Blood Cells
Source: Cells. 2022 Aug 9;11(16):2473. doi: 10.3390/cells11162473 (PMC9406338; doi:10.3390/cells11162473)
Supplement: Supplementary file 1 [file cells-11-02473-s001.zip › cells-1778946-supplementary.pdf]

Supplementary Material

Membrane properties of cultured red blood cells derived from human induced pluripotent stem cells

Running head: Membrane properties of iPSC-derived cultured RBCs

Claudia Bernecker [1], Eva M. Matzhold [1], Dagmar Kolb [2,3], Afrim Avdili [1], Lisa Rohrhofer [1], Annika Lampl [1], Martin Trötzmüller [4], Heike Singer [5], Johannes Oldenburg [5], Peter Schlenke [1] and Isabel Dorn [1]*

Supplementary Tables

Table S1: Human iPSC lines used in the study

iPSC	name	origin	reprogramming	reference
iPSC-1	PEB-iPSC	erythroblasts	Lentiviral<br>(OCT4, SOX2, KLF4, C-MYC)	[1]
iPSC-2	CD34-iPSC	cord blood CD34+ cells	Lentiviral<br>(OCT4, SOX2, KLF4, C-MYC)	[2]
iPSC-3	PEB-AL	erythroblasts	Episomal<br>(OCT3/4, SOX2, KLF4, LIN28, L-MYC)	
iPSC-4	CM1	erythroblasts	Episomal<br>(OCT4, SOX2, LIN28, KLF4, L-MYC)	[3]
iPSC-5	SANi-003A	erythroblasts	Episomal<br>(OCT4, SOX2, LIN28, C-MYC)	[4]

Table S2: Reagents (all from BioRad) used for RBC antigen expression analyses

Antigen	ID-card	Antibodies
cRBC suspension in Diluent 2 (LISS)		
ABO, D	DiaClon ABO/Rh for patients, monoclonal	
CcEe K	DiaClon Rh-Subgroups + K monoclonal	
C^w	DiaClon anti-C^w monoclonal	
Fy^a	ID-Card Fy^a	ID-Anti-Fy^a (human), Id-n 09210
Fy^b	ID-Card Fy^b	ID-Anti-Fy^b (human), Id-n 09310
M	DiaClon anti-M monoclonal	
N	DiaClon anti-N monoclonal	
S	LISS/Coombs	Anti-S (human), Id-n 19010
s	LISS/Coombs	Anti-s (human), Id-n 19110
cRBC suspension in Diluent 1 (Bromelin)		
k	Anti-k (human)	
Kp^a	Anti-Kp^a polyclonal	
Kp^b	Anti-Kp^b polyclonal	
Jk^a	DiaClon Anti-Jk^a monoclonal	
Jk^b	DiaClon Anti-Jk^b monoclonal	

Table S3: Cellular characteristics of cRBC_iPSCs compared to cRBC_adult, CB-derived nRETs, and PB-derived nRBCs. Generation of cRBC_adult from adult HSPCs and subsequent phenotyping was performed by our group as published [5]. All methods were identical to methods used for cRBC_iPSCs culturing. Cell diameter was measured microscopically. Mean corpuscular volume MCV [fl], mean corpuscular hemoglobin content (CHm) [pg] and mean corpuscular hemoglobin concentration (CHCm) [g/dl] were assessed by ADVIA analyzer [5]. Due to limited cell numbers ADVIA analyses were not performed with cRBC_iPSCs. The Hb composition was determined by HPLC analysis as published [1, 2]. (n.d. not determined; FC flow cytometry analysis)

	**cRBC_iPSC**	**cRBC_adult**	**CB-derived nRET**	**PB-derived nRBC**
**Phenotype flow cytometry (% positive cells)** [data derived from 5]				
GPA	>99	>99	>99	>99
Band3	>99	>99	>99	>99
CD36	21.5 ± 12.3	23.2 ± 5.4	31.6 ± 9.4	4.7 ± 1.7
CD49d	2.9 ± 2.1	11.3 ± 5.4	6 ± 1.6	n.d.
CD71	94.4 ± 3.3	80 ± 5.3	95 ± 3	1.2 ± 0.4
Thiazol orange	33.6 ± 16	41.8 ± 1.1	88 ± 4.2	2.6 ± 0.6
**Cell size, volume, Hb content**				
Diameter (µm)	11.2 ± 1.5	9.4 ± 0.2	8.1 ± 2.2	7.4 ± 0.8
MFI FSC (FC)	359,434 ± 23,355	340,900 ± 70,740	334,606 ± 18,539	294,483 ± 8,531
MFI SSC (FC)	339,976 ± 16,074	269,605 ± 47,145	324,167 ± 37,941	300,242 ± 16,997
MCV (fl) [5]	n.d.	128.7 ± 11.7	125.1 ± 5.7	89.3 ± 1,5
CHm (pg) [5]	n.d.	32.7 ± 1.3	35.2 ± 1	30.8 ± 1.5
CHCm (g/dL) [5]	n.d.	26.0 ± 2.2	28.3 ± 1.1	34.7 ± 1.5
Hemoglobin (mg/mL) (Drabkin staining)	205.6 ± 46.0	136.3 ± 24.7	201.4 ± 29.7	131.3 ± 17.9
% Hemoglobin (HPLC) [data derived from 1,2]	HbF 68.5 ± 2.7<br>HbA 3.3 ± 1.1<br>embHb 18.5 ± 1.8	HbF 9.1 ± 4.3<br>HbA 76.2 ± 7.8<br>embHb <1	HbF 87.3 ± 1.1<br>HbA 14.7 ± 5.1<br>embHb <1	HbF 3.3 ± 2.2<br>HbA 87.4 + 2.5<br>embHb <1
**Deformability and osmotic resistance (OR)**				
LORRCA EImax	0.44 ± 0.01	0.49 ± 0.01 [5]	0.53 ± 0.02	0.6 ± 0.01
Osmotoc resistance (% NaCl resulting in 50% hemolysis)	0.3	0.35 – 0.3 [5]	0.35 – 0.3	0.45 – 0.4

Supplementary Figure legends

Figure S1: Flow cytometry characterization of day 18 cRBC_iPSCs. Bars represent mean ± SD (n=18).

Figure S2: Maturation stage of cRBC_iPSCs determined by microscopy. Representative cytospin preparations obtained from nRBCs, nRETs, and cRBCs derived from the different iPSC lines, and stained with New methylene blue for the residual nucleic acid. (Scale bar 20 µm, magnification of 100× with oil).

Figure S3: Maturation stage of cRBC_iPSCs determined by flow cytometry. Representative flow cytometry analyses for Thiazole orange and CD71 obtained from nRBCs, nRET, and cRBCs derived from the different iPSC lines.

Figure S4: Representative microscopic images obtained from cell diameter measurements. Upper part: May-Gruenwald stained cytospin preparations (40fold magnification, scale bars represent 25 µm). Lower part: Magnification of marked section from the upper part. Cell diameters (white bars) were calculated using the EVOS M5000 software. At least 300 cells per slide were evaluated.

Figure S5: Mean immunofluorescence intensity (MFI) for Ankyrin, α-Spectrin, Band 3, and f-Actin. The MFI was calculated over the whole image field and quantified with the Nikon General Analysis 3 Software. No statistical differences were observed between cells from the different sources (n = 4 each).

Figure S6: Expression of blood group antigens by cRBC_iPSCs 1 (phenotyping). Expression of ABO, Rhesus, Kell, Kidd, Duffy, and MNSs antigens were evaluated using commercial accessible test systems for diagnostic in gel cards. The cRBC_iPSCs shown here are O Rh (D)

pos, ccD.Ee, Cw-, Kell-k+, Kpa-Kpb+, Jka+Jkb-, Fya-Fyb-, M+N+, S+s+. Controls (ctl) are negative.

Figure S7: Expression of blood group antigens by cRBC_iPSCs 5 (phenotyping). Expression of ABO, Rhesus, Kell, Kidd, Duffy, and MNSs antigens were evaluated using commercial accessible test systems for visual evaluation in gel cards. The cRBC_iPSCs shown here are O Rh (D) pos, CcD.ee, Cw-, Kell-k+, Kpa-Kpb+, Jka+Jkb+, Fya+Fyb-,M+N-, S-s+. Controls (ctl) are negative.

Supplementary Figures

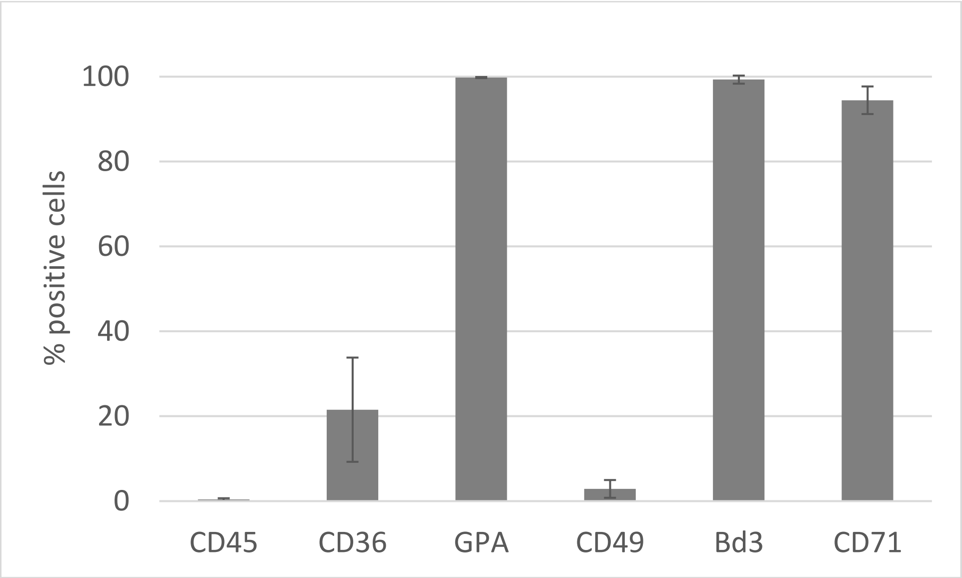

Figure S1: Flow cytometry characterization of day 18 cRBC_iPSCs.

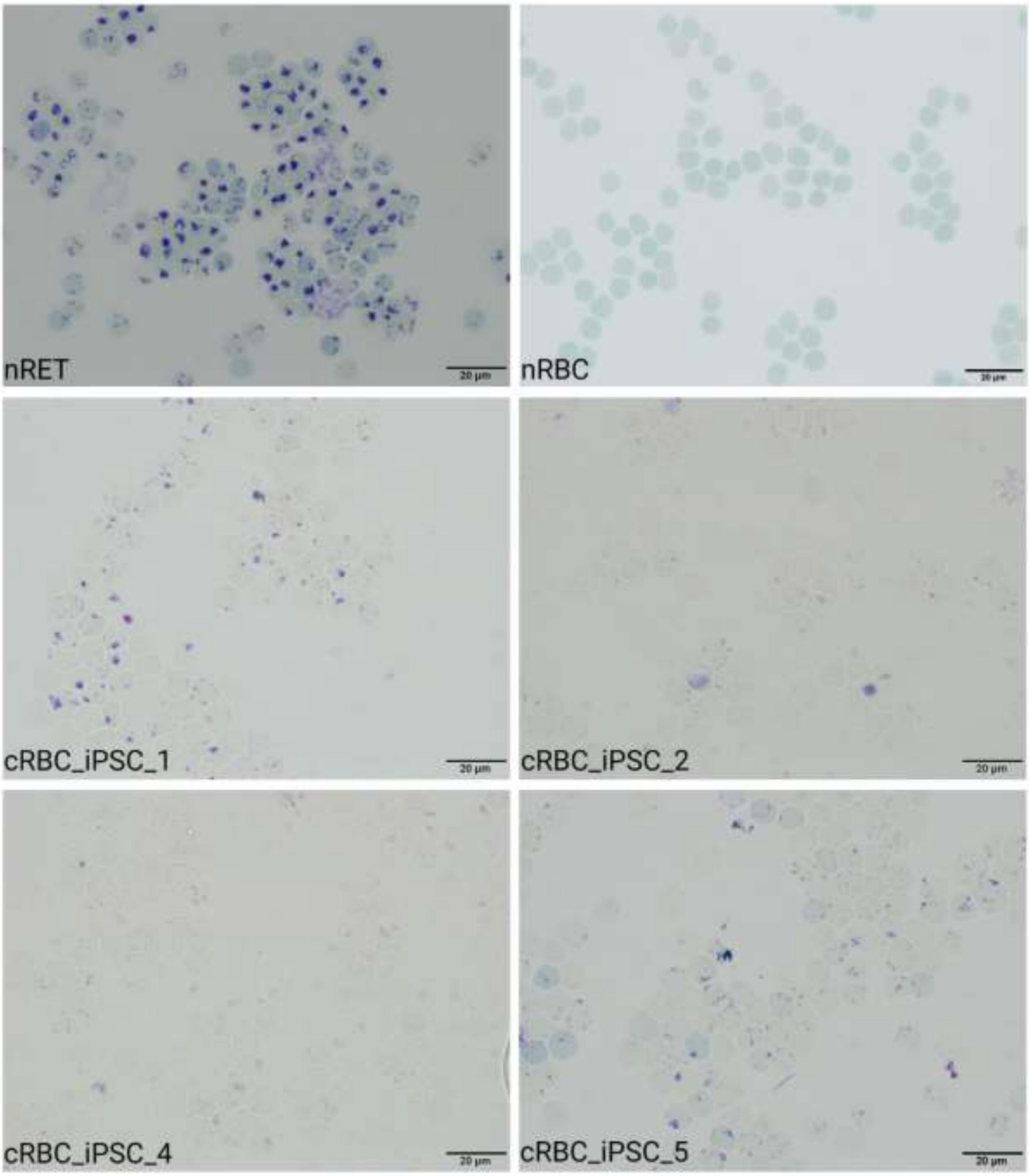

Figure S2: Maturation stage of cRBC_iPSCs determined by microscopy.

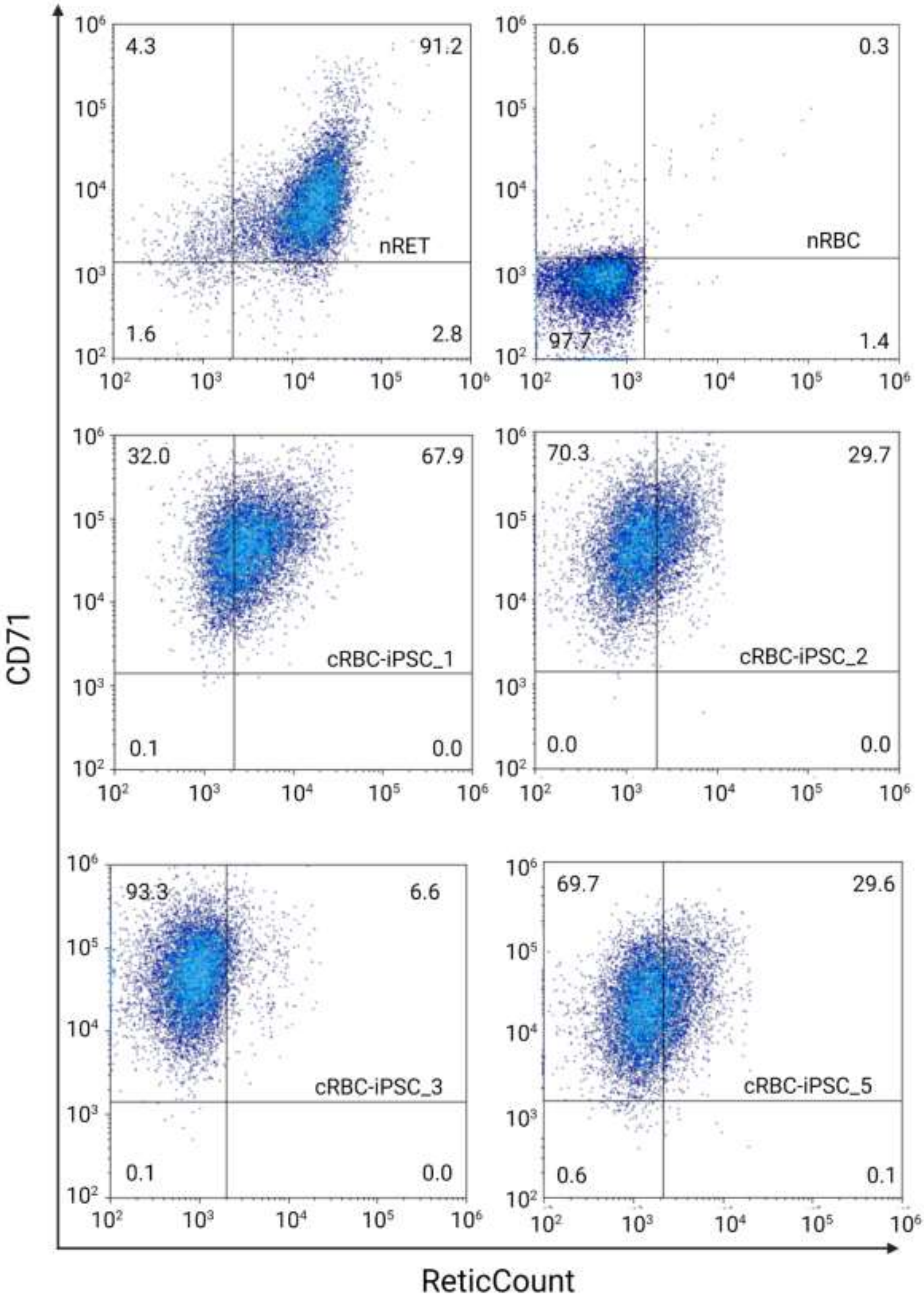

Figure S3: Maturation stage of cRBC_iPSCs determined by flow cytometry.

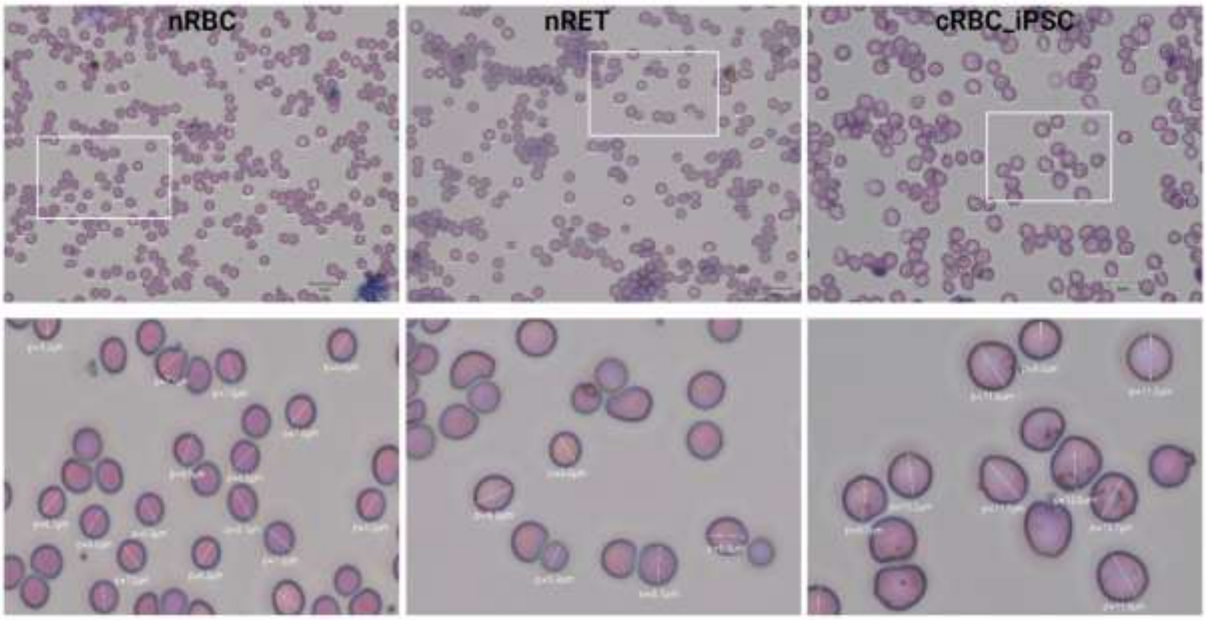

Figure S4: Representative microscopic images obtained from cell diameter measurements.

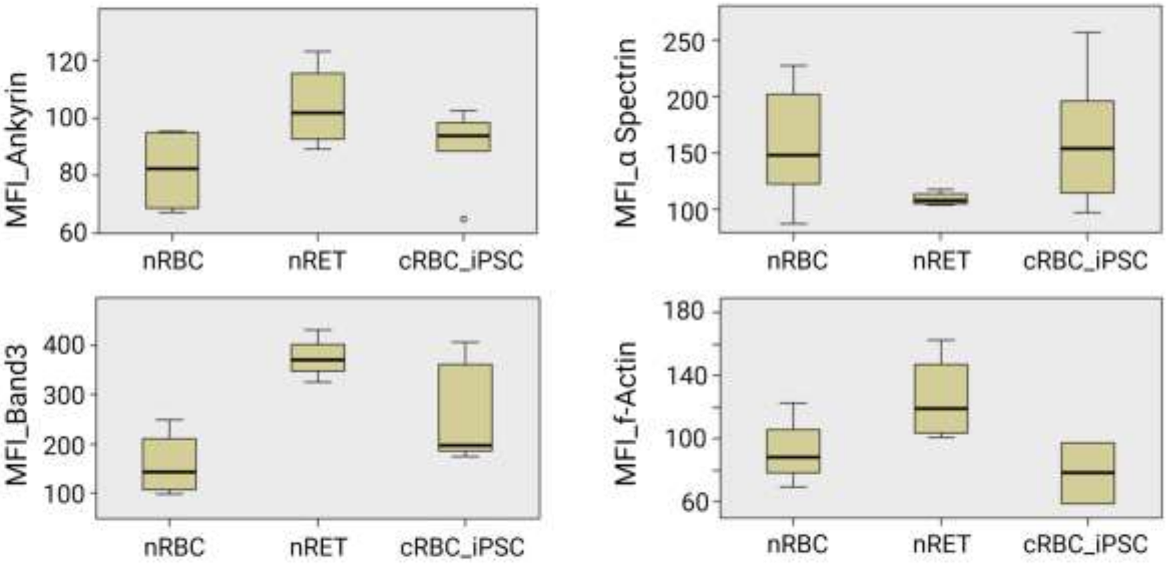

Figure S5: Mean immunofluorescence intensity (MFI) for Ankyrin, α-Spectrin, Band 3, and f-Actin.

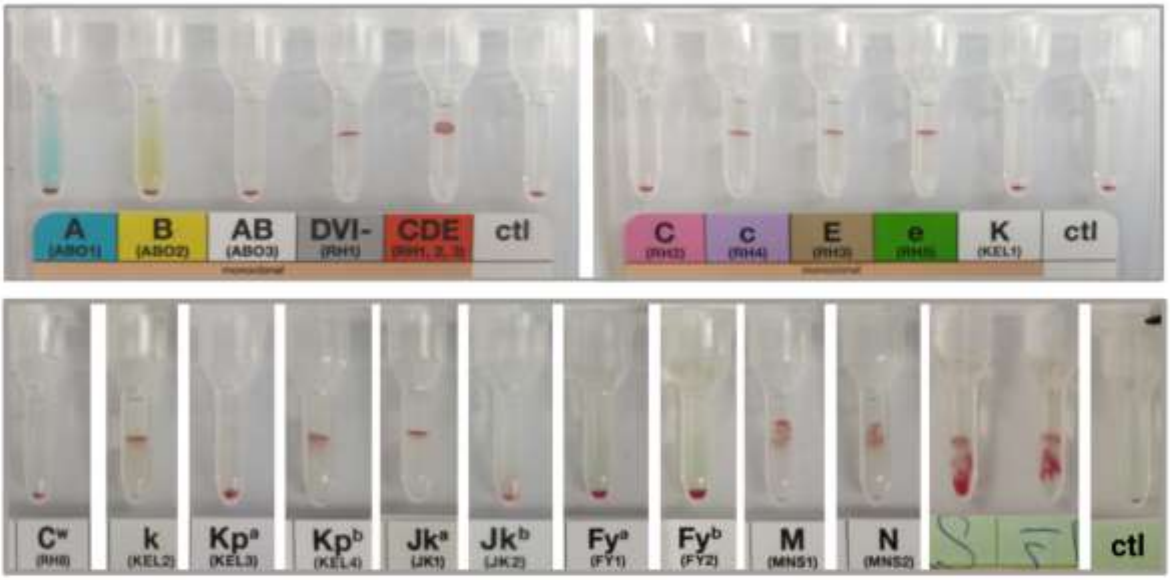

Figure S6: Expression of blood group antigens by cRBC_iPSCs 1 (phenotyping).

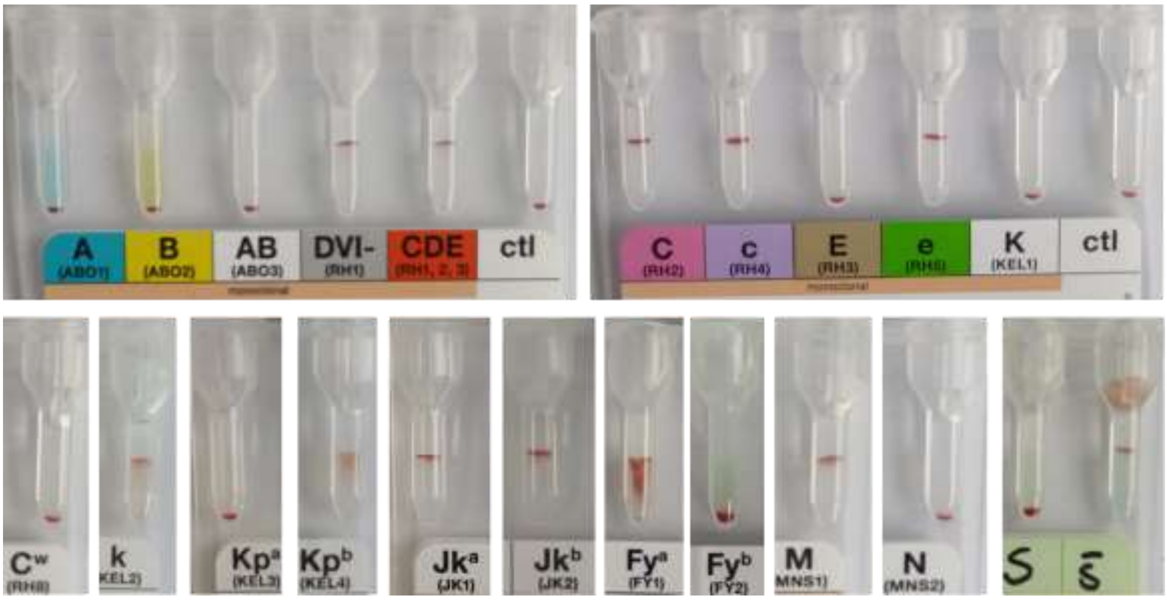

Figure S7: Expression of blood group antigens by cRBC_iPSCs 5 (phenotyping).

Supplementary References

1. Bernecker, C.; Ackermann, M.; Lachmann, N.; Rohrhofer, L.; Zaehres, H.; Arauzo-Bravo, M.J.; van den Akker, E.; Schlenke, P.; Dorn, I. Enhanced ex vivo generation of erythroid cells from human induced pluripotent stem cells in a simplified cell culture system with low cytokine support. *Stem cells and development* **2019**, *28*, 1540-1551, doi:10.1089/scd.2019.0132.
2. Dorn, I.; Klich, K.; Arauzo-Bravo, M.J.; Radstaak, M.; Santourlidis, S.; Ghanjati, F.; Radke, T.F.; Psathaki, O.E.; Hargus, G.; Kramer, J.*, et al.* Erythroid differentiation of human induced pluripotent stem cells is independent of donor cell type of origin. *Haematologica* **2015**, *100*, 32-41, doi:10.3324/haematol.2014.108068.
3. Jamil, M.A.; Singer, H.; Al-Rifai, R.; Nüsgen, N.; Rath, M.; Strauss, S.; Andreou, I.; Oldenburg, J.; El-Maarri, O. Molecular analysis of fetal and adult primary human liver sinusoidal endothelial cells: A comparison to other endothelial cells. *International journal of molecular sciences* **2020**, *21*, 7776
4. Varga, E.; Hansen, M.; Wust, T.; von Lindern, M.; van den Akker, E. Generation of human erythroblast-derived ipsc line using episomal reprogramming system. *Stem Cell Res* **2017**, *25*, 30-33, doi:10.1016/j.scr.2017.10.001.
5. Bernecker, C.; Kofeler, H.; Pabst, G.; Trotzmuller, M.; Kolb, D.; Strohmayer, K.; Trajanoski, S.; Holzapfel, G.A.; Schlenke, P.; Dorn, I. Cholesterol deficiency causes impaired osmotic stability of cultured red blood cells. *Frontiers in physiology* **2019**, *10*, 1529, doi:10.3389/fphys.2019.01529.
